# Assessment of the diagnostic efficacy of five non-invasive tests for MASLD: external validation utilizing data from two cohorts

**DOI:** 10.3389/fnut.2025.1571487

**Published:** 2025-05-22

**Authors:** Haoxuan Zou, Wen Pan, Xiaobin Sun

**Affiliations:** ^1^Department of Gastroenterology and Hepatology, The Affiliated Hospital of Southwest Jiaotong University, The Third People’s Hospital of Chengdu, Chengdu, Sichuan, China; ^2^Department of Health Management Center, The Hospital of Chengdu Office of People’s Government of Tibetan Autonomous Region, Chengdu, Sichuan, China

**Keywords:** MASLD, NITs, AUC, DCA, NRI, IDI

## Abstract

**Background:**

Numerous non-invasive tests (NITs) have been developed for non-alcoholic fatty liver disease (NAFLD) over the past few decades. However, their applicability to metabolic-associated steatotic liver disease (MASLD), as redefined and renamed by the recent Delphi Consensus Statement, necessitates further investigation. Consequently, this study aims to systematically evaluate the diagnostic efficacy of five clinically utilized NITs (FLI/FSI/ZJU/LAP/HSI) in assessing the risk of MASLD.

**Methods:**

The participants for this study were sourced from the Health Management Center at the Hospital of Chengdu Office of the Tibetan Autonomous Region, as well as from the National Health and Nutrition Examination Survey (NHANES) 2017–2020.3 cycle. The diagnostic efficacy of NITs was thoroughly evaluated and compared via methods such as the area under the curve (AUC), subgroup AUC, and clinical utility.

**Results:**

This study incorporated a total of 2,187 participants from the Health Management Center cohort and 5,524 participants from the NHANES cohort. In both cohorts, the FLI, FSI, LAP, ZJU, and HSI performed well in identifying those at high risk for MASLD. This effectiveness was consistently observed across various subgroups, including those defined by age, sex, race, overweight, hypertension, and diabetes status. Specifically, within the NHANES cohort, the FSI and FLI emerged as superior predictors of MASLD risk, with AUC values of 0.836 (95% CI: 0.826–0.847) and 0.835 (95% CI: 0.825–0.845), respectively. The difference in the AUC between these two NITs was not statistically significant (*p* > 0.05). In contrast, the ZJU, FLI, and FSI were more predictive of MASLD risk in the Health Management Center cohort. The AUC and 95% CI were: 0.815 (0.797–0.832), 0.810 (0.792–0.828), and 0.805 (0.787–0.823), respectively, and the difference in the AUC among them was not statistically significant (*p* > 0.05). The results remained the same when analyzed from the point of view of clinical utility, i.e., NRI, IDI, and DCA analyses were performed.

**Conclusion:**

Overall, the FLI, FSI, ZJU, LAP, and HSI continue to demonstrate significant diagnostic value, even when they are applied to the newly designated MASLD and are suitable for screening in high-risk populations.

## Introduction

Non-alcoholic fatty liver disease (NAFLD), a chronic liver disease linked to insulin resistance (IR), is now the most common liver condition globally, surpassing viral hepatitis (1–3). Major risk factors include high-calorie diets, sedentary lifestyles, obesity, metabolic syndrome, and diabetes, which are the major risk factors for NAFLD (4). The original disease terminology’s lack of focus on etiology and pathogenesis is increasingly problematic, hindering the screening, diagnosis, prevention, and management of fatty liver disease (5). As a result, in 2020, a global panel of 31 experts renamed NAFLD to metabolic dysfunction-associated fatty liver disease (MAFLD), highlighting metabolic dysfunction in its diagnostic criteria. However, MAFLD allows for the coexistence of multiple etiologies raising concerns about etiologic heterogeneity and potential stigmatization due to the term “fatty” (6, 7). Recently, a novel nomenclature for steatotic liver disease (SLD) was proposed. This new classification system introduces multiple subcategories with a rigorous delineation of etiologies. It replaces the original term NAFLD with metabolic dysfunction-associated steatotic liver disease (MASLD), thereby underscoring the significance of cardiometabolic risk factor (CMRF) while eliminating stigmatization and etiologic heterogeneity (8). Most MASLD patients are identified during physical exams and often lack specific symptoms, making costly and invasive diagnostic tools such as magnetic resonance imaging, vibration-controlled transient elastography (VCTE), and liver biopsy less acceptable and accessible. Thus, simple, inexpensive, and reproducible non-invasive tests (NITs) based on routine body composition and blood tests are more suitable for screening high-risk individuals, especially in primary care settings.

In recent decades, numerous NITs have been developed for the diagnosis of NAFLD. Bedogni et al. (9) developed the fatty liver index (FLI), which incorporates body mass index (BMI), waist circumference (WC), triglyceride (TG), and *γ*-glutamyl transpeptidase (GGT) levels. The model demonstrated an area under the curve (AUC) of 0.84, with a 95% confidence interval (CI) ranging from 0.81 to 0.87 (9). The Framingham steatosis index (FSI) was developed by Long et al. utilizing data from 1,181 participants of the Framingham Third Generation Cohort, which incorporated variables such as age, sex, BMI, TG, hypertension, diabetes, and the alanine aminotransferase (ALT) to aspartate aminotransferase (AST) ratio, yielding an AUC of 0.845 (10). The lipid accumulation product (LAP) (11), initially developed to estimate excessive lipid elevation based on BMI and TG, has subsequently been employed in the diagnosis of NAFLD. The Zhejiang University index (ZJU) was founded by Wang et al. (12). The developed formula, which incorporates BMI, fasting plasma glucose (FPG), TG, and the ALT/AST ratio, demonstrated a diagnostic value of 0.822 (95% CI: 0.810–0.834) for NAFLD within the training cohort and achieved an AUC of 0.826 (95% CI: 0.815–0.838) (12). The hepatic steatosis index (HSI) was originally a simple screening tool constructed by Lee et al. (13) for NAFLD, consisting of the ALT/AST ratio, BMI, diabetes status, and gender, with an AUC and 95% CI of 0.812 (0.801–0.824).

These five NITs are among the most widely utilized. Certain guidelines have recommended the use of these NITs to estimate the prevalence of NAFLD in epidemiological studies. However, it remains uncertain whether these NITs retain their diagnostic accuracy for the recently reclassified MASLD. To address this, we conducted an external validation of the diagnostic performance of the FLI, FSI, LAP, ZJU, and HSI for MASLD using data from two independent cohorts within the National Health and Nutrition Examination Survey (NHANES) and the Health Management Center at the Hospital of Chengdu Office of the Tibetan Autonomous Region.

## Materials and methods

### Data sources

This study used data from NHANES 2017–2020 March, a survey by the National Center for Health Statistics (NCHS) that employs a stratified multistage sampling design to represent the United States residents. Additionally, another cohort was sourced from the Health Management Center at the Hospital of Chengdu Office of the Tibetan Autonomous Region (2022.1–2023.12). The study was approved by the hospital’s Ethics Committee and followed the ethical guidelines of the Declaration of Helsinki. Additionally, this study followed the same methodology outlined in the Multivariable Predictive Model for Individual Prognosis or Diagnosis (TRIPOD) guidelines (14).

### Clinical assessment

The NHANES and Health Management Center cohorts were the main sources for all variables, including demographics, anthropometrics, lab factors, and comorbidities, as detailed in the Supplementary material. Formulas for NITs [FLI (9), FSI (10), LAP (11), ZJU (12), and HSI (13)] are also provided in the Supplementary material.

### Definition of MASLD

Hepatic steatosis can be precisely identified through the application of controlled attenuation parameters (CAP) via VCTE. This technique employs a 3.5 MHz ultrasound frequency to penetrate the liver parenchyma, where the degree of ultrasound attenuation is directly proportional to the lipid content within hepatocytes, thereby facilitating the assessment of the severity of hepatic steatosis (15–17). Previous studies in the literature have established that a CAP threshold of ≥274 dB/m is indicative of significant hepatic steatosis (18). MASLD was defined as the presence of significant liver steatosis and at least one CMRF, excluding those with excessive alcohol intake (>140 grams/week for females, >210 grams/week for males) or other causes of liver steatosis (details are available in the Supplementary material) (8).

### Statistical analyses

Statistical analyses were conducted via R 4.3.2, with significance set at *p* < 0.05. Continuous variables are presented as mean ± standard deviation (SD) and were compared using Student’s t test or the Mann–Whitney *U* test. Categorical variables are shown as percentages and were compared via the *χ*^2^ test. The sensitivity (SEN) and specificity (SPE) of each possible cutoff value of the non-invasive tests were used to create receiver operating characteristic (ROC) curves, and the optimal cutoff value was determined on the basis of the principle of maximizing the Youden index, as were the corresponding AUC, SEN, SPE, positive predictive value (PPV), and negative predictive value (NPV). The Delong method was used to check for significant differences in the AUC between non-invasive tests (19). Furthermore, the present study determined optimal cutoff values utilizing the Youden index (20). Additionally, the study also employed integrated discrimination improvement (IDI), net weight classification index (NRI), and decision curve analysis (DCA) (21–23) to further evaluate its clinical utility.

## Results

### Characteristics of the participants

**Figure 1:**
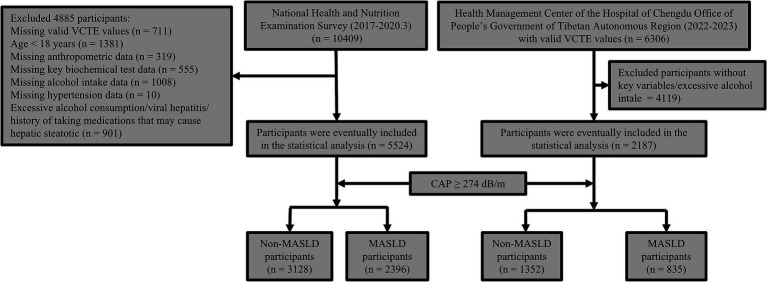
Diagram of the study design flow.

From the NHANES 2017–2020 March cohort, 10,409 participants were initially considered, but after those with missing data were excluded, 5,524 were eligible for the study. Among them, 2,396 met the diagnostic criteria for MASLD, resulting in a prevalence of 43.37%. Similarly, from the Health Management Center at the Hospital of Chengdu Office of the Tibetan Autonomous Region cohort (2022.1–2023.12), 6,306 participants were considered, with 2,187 eligible after exclusions. Of these, 835 met the MASLD criteria, yielding a prevalence of 38.18%. The inclusion and exclusion criteria are outlined in Figure 1. Table 1 reveals that participants with MASLD were older, predominantly male, had a higher proportion of diabetes and hypertension, had higher levels of WC, BMI, CAP, liver stiffness measurements (LSM), and had elevated indicators of glucose, lipid, and liver enzyme levels, but lower high-density lipoprotein cholesterol (HDL) levels than did those without MASLD in both the NHANES and Health Management Center cohorts. NITs for predicting MASLD risk, including the FLI, FSI, LAP, ZJU, and HSI, were significantly higher in the MASLD group (all *p* < 0.001).

**Table 1:** Baseline characteristics of participants with or without MASLD assessed by VCTE in the NHANES and the Health Management Center cohorts.

Variables	NHANES cohort			Health Management Center cohort		
	Non-MASLD (*n* = 3,128)	MASLD (*n* = 2,396)	*P-* value	Non-MASLD (*n* = 1,352)	MASLD (*n* = 835)	*p*-value
Age (years)	46.04 ± 18.86	52.17 ± 16.41	<0.001	44.50 ± 11.65	46.73 ± 11.18	<0.001
Male (%)	1,443 (46.13%)	1,346 (56.18%)	<0.001	799 (59.10%)	628 (75.21%)	<0.001
Race (%)			<0.001			–
Non-Hispanic Black	855 (27.33%)	487 (20.33%)		–	–	
Non-Hispanic White	1,140 (36.45%)	899 (37.52%)		–	–	
Other Hispanic	633 (20.24%)	672 (28.05%)		–	–	
Non-Hispanic Asian	344 (11.00%)	219 (9.14%)		–	–	
Other races	156 (4.99%)	119 (4.97%)		–	–	
WC (cm)	92.73 ± 14.37	111.04 ± 15.44	<0.001	83.25 ± 9.94	93.30 ± 9.10	<0.001
BMI (kg/m^2^)	26.98 ± 5.86	33.82 ± 7.16	<0.001	24.24 ± 2.99	27.61 ± 3.00	<0.001
CAP (dB/m)	220.20 ± 36.25	323.29 ± 36.15	<0.001	225.52 ± 32.90	313.48 ± 28.98	<0.001
LSM (kPa)	5.07 ± 3.46	6.97 ± 6.11	<0.001	4.60 ± 2.10	5.19 ± 1.97	<0.001
ALT (U/L)	18.26 ± 13.41	26.17 ± 17.87	<0.001	32.01 ± 32.46	45.35 ± 33.97	<0.001
AST (U/L)	20.06 ± 9.15	22.34 ± 12.32	<0.001	23.88 ± 13.17	27.24 ± 15.78	<0.001
ALP (U/L)	74.56 ± 26.59	80.53 ± 24.49	<0.001	80.48 ± 35.09	84.89 ± 25.77	0.002
GGT (U/L)	24.41 ± 35.31	36.13 ± 42.67	<0.001	44.21 ± 67.30	66.00 ± 81.11	<0.001
FPG (μmol/L)	5.25 ± 1.52	6.09 ± 2.41	<0.001	5.03 ± 1.34	5.45 ± 1.49	<0.001
TG (μmol/L)	1.25 ± 0.78	1.91 ± 1.32	<0.001	1.42 ± 0.91	2.03 ± 1.44	<0.001
TC (μmol/L)	4.72 ± 1.02	4.85 ± 1.06	<0.001	4.73 ± 0.89	5.01 ± 0.91	<0.001
HDL (μmol/L)	1.46 ± 0.39	1.23 ± 0.34	<0.001	1.38 ± 0.31	1.27 ± 0.25	<0.001
LDL (μmol/L)	2.69 ± 0.89	2.75 ± 0.96	0.013	2.83 ± 0.74	3.12 ± 0.73	<0.001
Hypertension	832 (26.60%)	1,121 (46.79%)	<0.001	156 (11.54%)	202 (24.19%)	<0.001
Diabetes	334 (10.68%)	723 (30.18%)	<0.001	61 (4.51%)	100 (11.98%)	<0.001
FLI	39.34 ± 29.91	77.33 ± 22.83	<0.001	32.34 ± 24.02	61.62 ± 22.15	<0.001
FSI	−1.78 ± 1.48	0.37 ± 1.72	<0.001	−1.96 ± 1.20	−0.50 ± 1.34	<0.001
LAP	41.60 ± 35.63	93.26 ± 70.60	<0.001	31.33 ± 26.85	60.22 ± 46.27	<0.001
ZJU	37.23 ± 6.79	46.12 ± 8.09	<0.001	35.32 ± 4.18	40.40 ± 4.34	<0.001
HSI	35.40 ± 6.98	44.42 ± 8.13	<0.001	35.30 ± 5.83	41.18 ± 5.52	<0.001

Continuous variables are shown as mean ± SD and compared by Student’s *t*-test or Mann–Whitney *U*-test. Categorical values are shown as % and compared using the *χ*^2^ test. MASLD, metabolic dysfunction-associated steatotic liver disease; NHANES, National Health and Nutrition Examination Survey; VCTE, vibration-controlled transient elastography; CAP, controlled attenuation parameter; LSM, liver stiffness measurements; BMI, body mass index; WC, waist circumference; FPG, fasting plasma glucose; ALT, alanine aminotransferase; AST, aspartate aminotransferase; GGT, γ-glutamyl transpeptidase; ALP, alkaline phosphatase; TC, total cholesterol; TG, triglyceride; HDL, high-density lipoprotein cholesterol; LDL, low-density lipoprotein cholesterol; FLI, fatty liver index; FSI, Framingham steatosis index; HSI, hepatic steatosis index; ZJU, Zhejiang University index; LAP, lipid accumulation product.

### Evaluation of NITs for their efficacy in discriminating the risk of MASLD within the NHANES cohort

**Figure 2:**
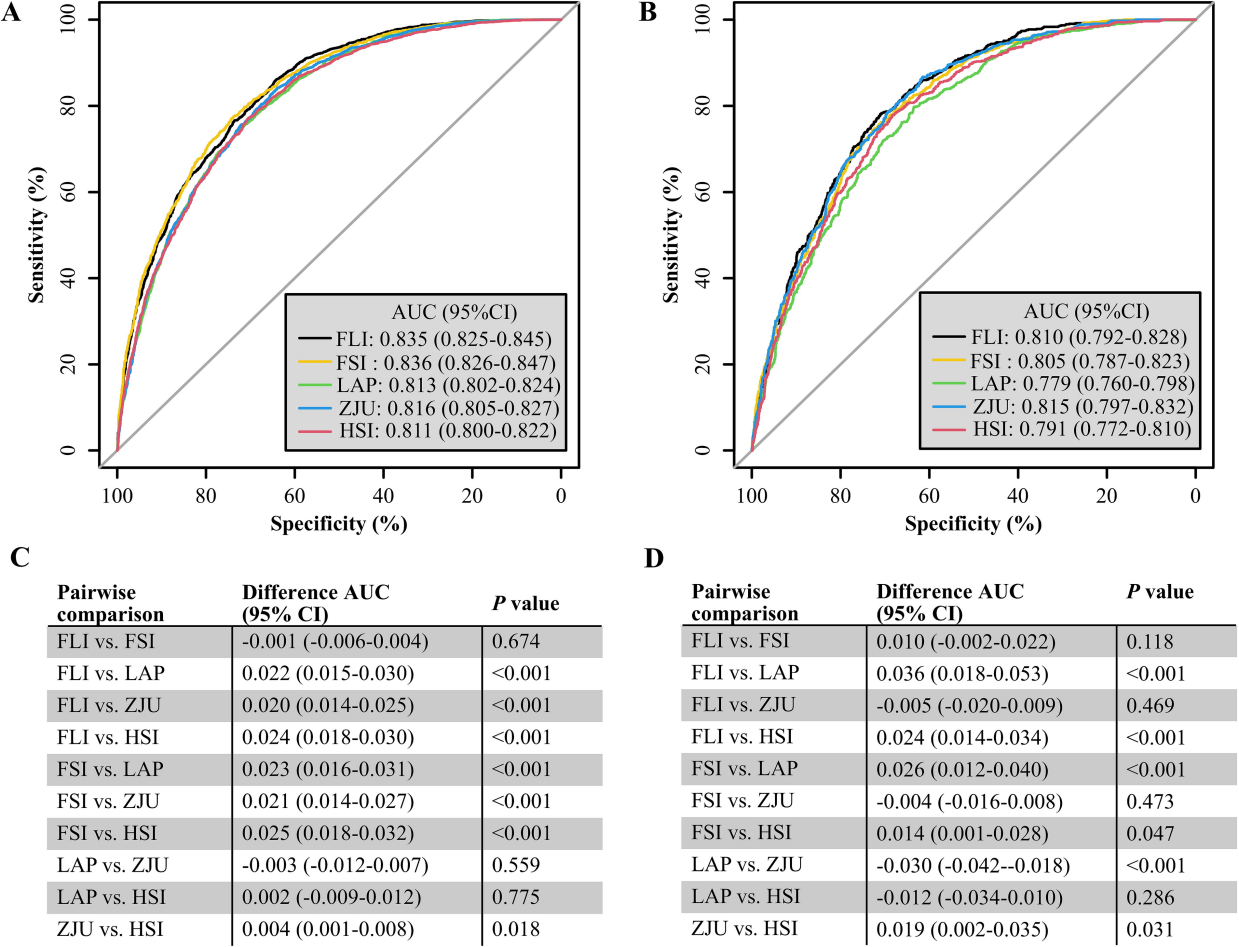
Receiver operating characteristic (ROC) curves for predicting MASLD in the NHANES **(A)** and Health Management Center **(B)** cohorts, with specificity on the *x*-axis and sensitivity on the *y*-axis. The DeLong method for the difference in the AUC of predicting MASLD by each NIT in the NHANES **(C)** and the Health Management Center cohorts **(D)**.

**Figure 3:**
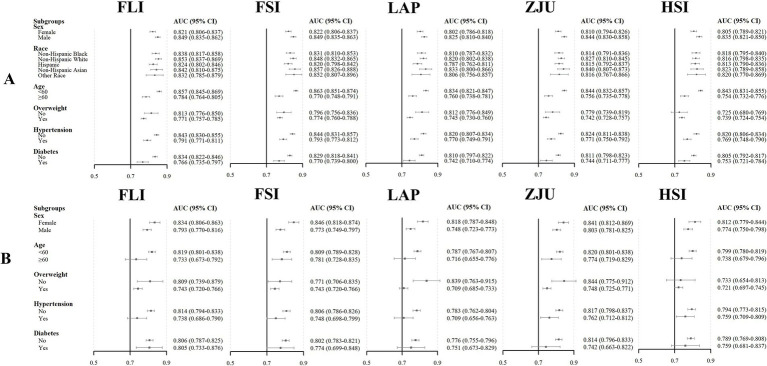
Area under the curve (AUC) and 95% CI for NITs to detect MASLD risk in different subgroups of the NHANES **(A)** and Health Management Center **(B)** cohorts.

**Figure 4:**
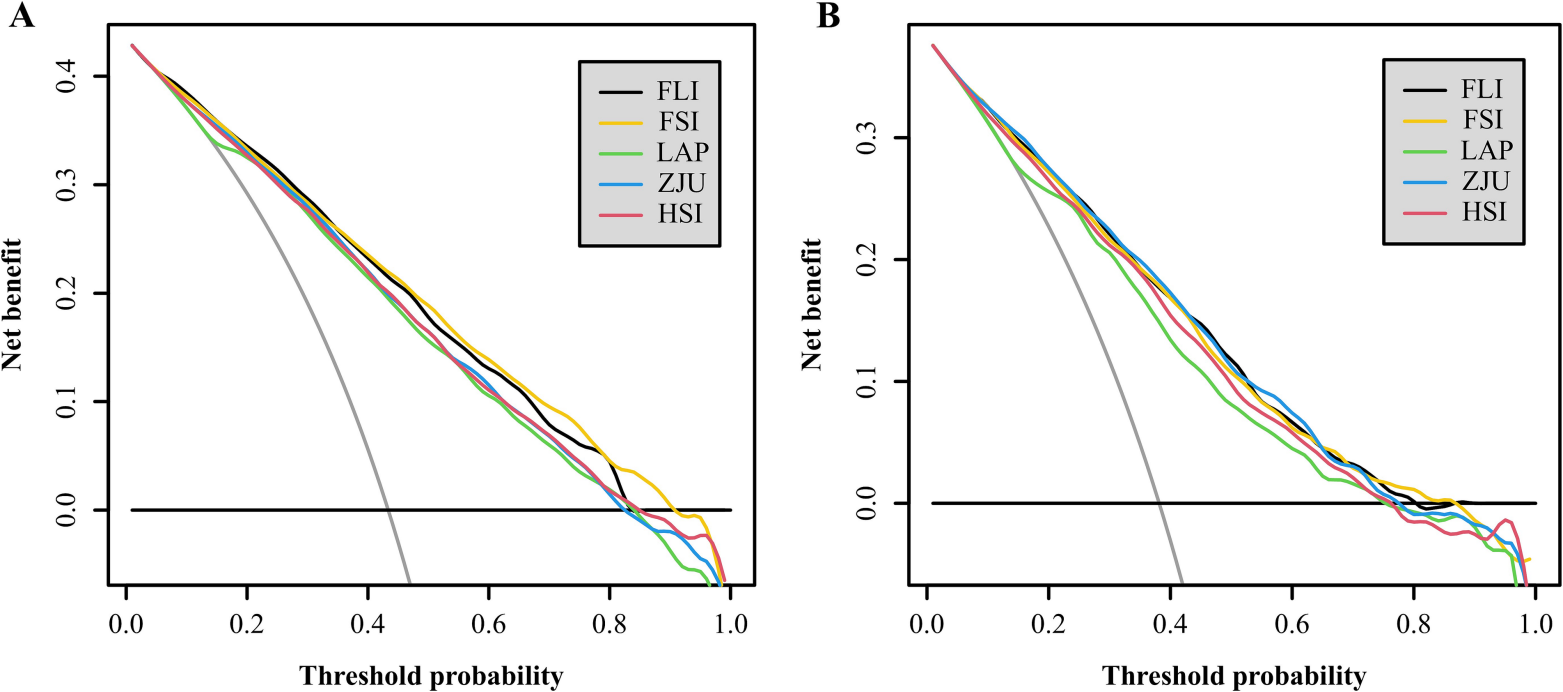
The clinical utility of the NITs for determining MASLD risk was assessed via DCA in the NHANES **(A)** and Health Management Center **(B)** cohorts, with the *x*-axis showing the threshold probability and the *y*-axis indicating the net benefits.

The AUC values for the five NITs used to assess the risk of MASLD were calculated and compared. All NITs demonstrated great diagnostic efficacy, with AUC values exceeding 0.800. The FSI exhibited the highest AUC of 0.836, with a 95% CI ranging from 0.826 to 0.847. This was followed by the FLI with an AUC of 0.835 (95% CI: 0.825–0.845), the ZJU with an AUC of 0.816 (95% CI: 0.805–0.827), the LAP with an AUC of 0.813 (95% CI: 0.802–0.824), and the HSI with an AUC of 0.811 (95% CI: 0.800–0.822) (Figure 2A). Detailed metrics including the SEN, SPE, PPV, NPV, and optimal cutoff values for each NIT are presented in Table 2. After conducting a statistical analysis to compare the differences in the AUC for each NIT, it was determined that the difference in the AUC between the FSI and the FLI was not statistically significant (*p* = 0.674). However, these indices demonstrated superior performance compared with other NITs (Figure 2C). The participants were stratified into subgroups based on gender, age, the presence of hypertension, diabetes, and BMI to evaluate the diagnostic efficacy of NITs (Figure 3A). Subgroup analyses revealed that the AUC for the FSI was highest among males, females, non-Hispanic Asians, individuals of other races, participants younger than 60 years, and those classified into the overweight, non-hypertensive, hypertensive, and diabetic subgroups. Conversely, the AUC for the FLI was highest among non-Hispanic Blacks, non-Hispanic Whites, Hispanics, participants older than 60 years, those classified as non-overweight, and non-diabetic subgroups (Supplementary Tables 2–6). Furthermore, to comprehensively assess the clinical utility of the five NITs, we calculated the NRI and IDI values among the NITs. The results indicated that the differences in the NRI and IDI values between the FSI and the FLI were not statistically significant. However, the NRIs and IDIs between the FSI/FLI and the other NITs were greater than zero and demonstrated statistical significance (Supplementary Table 1). In addition, this study employed DCA to evaluate the clinical utility of NITs. As illustrated in Figure 4A, the findings revealed that the maximum net benefit for all NITs reached 0.428. Notably, the FSI demonstrated the broadest threshold range for net benefit exceeding zero, spanning from 0.01 to 0.90.

**Table 2:** Performance assessment of the NITs for the prediction of MASLD risk.

NITs	AUC (95% CI)	SEN (95% CI)	SPE (95% CI)	PPV (95% CI)	NPV (95% CI)	Cutoff value
NHANES cohort						
FLI	0.835 (0.825–0.845)	0.861 (0.847–0.874)	0.642 (0.625–0.659)	0.648 (0.632–0.665)	0.857 (0.843–0.872)	48.693
FSI	0.836 (0.826–0.847)	0.776 (0.760–0.793)	0.737 (0.721–0.752)	0.693 (0.676–0.711)	0.811 (0.797–0.826)	−0.973
LAP	0.813 (0.802–0.824)	0.748 (0.731–0.765)	0.722 (0.706–0.738)	0.673 (0.656–0.691)	0.789 (0.774–0.804)	50.963
ZJU	0.816 (0.805–0.827)	0.826 (0.811–0.842)	0.654 (0.638–0.671)	0.647 (0.630–0.664)	0.831 (0.816–0.846)	38.847
HSI	0.811 (0.800–0.822)	0.772 (0.755–0.789)	0.704 (0.688–0.720)	0.667 (0.649–0.684)	0.801 (0.786–0.816)	38.285
Health Management Center cohort						
FLI	0.810 (0.792–0.828)	0.867 (0.844–0.890)	0.615 (0.589–0.641)	0.582 (0.555–0.609)	0.882 (0.862–0.903)	35.727
FSI	0.805 (0.787–0.823)	0.744 (0.714–0.773)	0.729 (0.705–0.752)	0.629 (0.598–0.659)	0.822 (0.800–0.843)	−1.307
LAP	0.779 (0.760–0.798)	0.798 (0.770–0.825)	0.635 (0.609–0.660)	0.574 (0.546–0.603)	0.835 (0.813–0.858)	31.810
ZJU	0.815 (0.797–0.832)	0.781 (0.753–0.809)	0.711 (0.687–0.735)	0.625 (0.596–0.654)	0.840 (0.819–0.861)	37.171
HSI	0.791 (0.772–0.810)	0.778 (0.750–0.807)	0.683 (0.659–0.708)	0.603 (0.574–0.632)	0.833 (0.811–0.855)	36.953

NITs, non-invasive tests; AUC, area under the receiver operating characteristic curve; SPE, specificity; SEN, sensitivity; NPV, negative predictive value; PPV, positive predictive value.

In conclusion, the findings indicate that within the NHANES cohort, the FLI, FSI, LAP, ZJU, and HSI all demonstrate significant predictive value for the risk assessment of MASLD. Among these, the FSI and FLI exhibit superior predictive capabilities.

### Assessing the efficacy of NITs in identifying MASLD risk in the Health Management Center cohort

Among the five NITs evaluated, the AUC values for the ZJU, FLI, and FSI surpassed 0.8. Specifically, the ZJU presented the highest AUC value of 0.815, with a 95% CI ranging from 0.797 to 0.832. This was followed by the FLI [AUC 0.810 (95% CI: 0.792–0.828)], and FSI [AUC 0.805 (95%CI: 0.787–0.823)]. In contrast, the HSI and LAP demonstrated lower AUC values of 0.791 (95% CI: 0.772–0.810) and 0.779 (95% CI: 0.760–0.798), respectively (Figure 2B). Statistical tests of AUC differences revealed that the differences in the ZJU, FLI, and FSI were not statistically significant, but all were better than those in the HSI and the LAP (Figure 2D). According to the subgroup analyses, ZJU had the highest AUC in males, those under 60 years, both overweight and non-overweight, hypertensive and non-hypertensive groups, and non-diabetics individuals. The FSI had the highest AUC in females and those aged 60 years or older, whereas the FLI had the highest AUC in diabetics (Figure 3B and Supplementary Tables 7–11). Furthermore, as shown in Supplementary Table 1, the diagnostic value of the ZJU, FSI, and FLI for MASLD remained consistent across the NRI and IDI assessments. Consistently, as presented in Figure 4B, the DCA curves indicate a consistent maximum net benefit of 0.376 for the ZJU, FSI, and FLI, with the FSI exhibiting the widest threshold probability range of 0.01–0.87.

Overall, on the basis of the above findings, the ZJU, FSI and FLI were strong predictors of MASLD in the Health Management Center at the Hospital of Chengdu Office of the Tibetan Autonomous Region.

## Discussion

Given the high global prevalence of NAFLD and the limited accessibility and high cost of conventional diagnostic methods such as imaging and liver biopsy, numerous NITs for NAFLD have been developed over the past decade, such as the FLI (9), FSI (10), LAP (11), ZJU (12), and HSI (13). These NITs serve as alternatives to traditional imaging or histological diagnosis, thereby enhancing the feasibility of population-based screening for NAFLD. NAFLD has experienced two nomenclatural changes in recent years, accompanied by modifications in its diagnostic criteria. Consequently, further investigation is warranted to ascertain whether these alterations have impacted the diagnostic efficacy of the associated NITs. Against this background, externally validated the diagnostic value of the mentioned NITs for MASLD in two cohorts, assessing the AUC, subgroups of AUC, NRI, IDI, and DCA. The results indicated good diagnostic value overall, with the FLI/FSI performing better in the NHANES cohort, and the ZJU/FLI/FSI showing superior diagnostic ability in the Health Management Center cohort. Furthermore, this study identified that within both the overweight and non-overweight subgroups, the AUC for most NITs was greater in the non-overweight subgroup compared to the overweight subgroup in both the NHANES and Health Management Center cohorts. These findings are consistent with those reported in previous studies (24–26). Additionally, a further analysis of gender distribution within the non-overweight population revealed a higher representation of women than men in both cohorts (female: 53.13 and 54.71%, respectively). It is posited that this phenomenon may be attributed to the reliance on BMI as the primary criterion for defining non-overweight status. Typically, women possess higher levels of subcutaneous and visceral adiposity, which may not be accurately captured by BMI alone, thereby rendering it an incomplete measure of adiposity (27, 28). Furthermore, existing research on non-obese NAFLD indicates a heightened susceptibility to metabolic disorders among non-obese individuals (29, 30). In conjunction with the subgroup analysis presented in this study, it is suggested that the risk of MASLD in non-overweight individuals warrants increased scholarly attention.

The externally validated articles on the NITs in this study demonstrated good diagnostic value for NAFLD/MAFLD. The FLI has been validated as possessing significant diagnostic value for NAFLD/MAFLD across various cohorts and has been unanimously endorsed by expert consensus as the NIT for screening NAFLD/MAFLD (7, 31). In a Netherlands cohort comprising 2,652 middle-aged and older adults, the AUC for the diagnosis of NAFLD was 0.813, with a 95% CI of 0.797–0.830 (32). Similarly, in a Chinese cohort of 8,626 individuals from Shanghai, the AUC for diagnosing NAFLD was 0.834, with a 95% CI of 0.825–0.842 (33). Furthermore, numerous external validations for MAFLD have demonstrated that the AUC for FLI ranges from 0.791 to 0.879, thereby maintaining a high diagnostic value (24, 25, 34–36). As for the FSI, in another study of 1,301 Korean health check-ups in which hepatic steatosis was diagnosed by magnetic resonance imaging, the AUC for the diagnosis of NAFLD by the FSI was 0.70 (95% CI 0.66–0.73) (37). In a separate external validation of NITs for NAFLD in a Chinese population, the AUC and 95% CI for FSI were 0.85 (0.84–0.86) (26). A study utilizing data from the NHANES 2017–2018, which encompassed 1866 participants, demonstrated that among individuals diagnosed with NAFLD or MAFLD using VCTE, the AUC and 95% CI for the FSI were 0.811 (0.791–0.832) and 0.833 (0.815–0.852), respectively (25). Overall, the diagnostic efficacy of FSI is deemed satisfactory. As for the LAP, a meta-analysis encompassing 16 studies with a total of 96,101 participants demonstrated that the pooled sensitivity and specificity of the LAP index for screening NAFLD were 94 and 85%, respectively (38). Furthermore, the AUC of the LAP, which possesses the simplest calculation formula among the five NITs discussed in this study, demonstrates satisfactory diagnostic performance for NAFLD and MAFLD. Notably, the AUC of the LAP for diagnosing NAFLD/MAFLD exceeded 0.799 in all subsequent external validation studies of NITs (24, 25, 35, 39). In external validation studies, ZJU demonstrated robust diagnostic value for both NAFLD and MAFLD (24, 26, 40, 41), not only within the Asian population but also satisfactorily within the U.S. population (24, 25, 42). Besides, in more than a decade of validation, the HSI has also demonstrated satisfactory diagnostic ability for NAFLD/MAFLD, across different ethnicities in different countries (24, 25, 34–36).

Metabolic-associated steatotic liver disease is a complex condition influenced by a combination of metabolic, genetic, and environmental factors (4). To date, the precise mechanisms underlying its pathogenesis remain incompletely understood. The prevailing hypothesis is the “multiple-hit” theory, which posits that MASLD arises from a confluence of genetic predispositions-such as variations in the transmembrane 6 superfamily member 2 gene and the patatin-like phospholipase domain-containing protein 3 gene-as well as epigenetic and other contributing factors, including IR, lipotoxicity, oxidative stress, mitochondrial dysfunction, and endoplasmic reticulum stress (43, 44). IR facilitates the translocation of free fatty acids (FFAs) to the liver via multiple pathways, contributing to lipotoxicity when the levels of FFAs surpass the oxidative capacity of cellular mitochondria. This lipotoxicity impairs insulin signaling, thereby inducing oxidative stress and leading to intrahepatic steatosis. As the inflammatory process escalates, it further promotes the progression of fibrosis and, in uncontrolled cases, may result in cirrhosis and/or hepatocellular carcinoma (HCC) (45–47). All NITs in this study incorporated metrics like BMI, WC, TG, and FPG, which are closely linked to IR (48, 49). Furthermore, BMI and WC, which serve as indicators of obesity, are associated with an elevated risk of progression in MASLD (50). A prospective study utilizing paired liver biopsies demonstrated that weight gain exceeding 5 kg during the follow-up period exacerbated hepatic fibrosis (51). In patients with baseline compensated cirrhosis, being overweight or obese heightens the risk of clinical decompensation. Moreover, obesity markedly increases the risk of developing MASLD-related HCC and is associated with increased HCC-related mortality (52). In MASLD, elevated liver enzymes typically show higher ALT than AST levels (53, 54). ALT, mainly in hepatocyte cytoplasm, signals hepatocyte injury, while AST, found in both mitochondria and cytoplasm, suggests more severe liver damage. The high ALT/AST ratio in early MASLD stages may result from IR interacting with oxidative stress and lipotoxicity, leading to fat buildup and inflammation in hepatocytes, thus raising ALT levels (43, 44). Studies indicate that the ALT/AST ratio independently correlates with hepatic steatosis (55–57) and is a better predictor of it than ALT alone (10). Similar to the elevation of the alanine/glutamine ratio, GGT may also increase with the development of MASLD (53). Oxidative stress plays an important role in the disease progression of MASLD by generating large amounts of reactive oxygen species (ROS) through multiple pathways (43, 44). Glutathione (GSH) is an important antioxidant and scavenger of ROS inside and outside human cells (58). As GSH reactivity is elevated, GGT, an enzyme that cleaves *γ*-glutamyl residues in GSH to cysteine-glycine, is also induced to be elevated (59). Consequently, variables indicative of IR, cardiometabolic risk, and liver enzymes which are incorporated into non-invasive indices such as the FLI, the FSL, and the ZJU, among others, may elucidate the efficacy of these indices in identifying patients at elevated risk for MASLD.

The present study possesses several strengths. Firstly, it includes a substantial sample size of 7,711 participants drawn from two distinct cohorts, thereby enhancing the reliability and generalizability of the findings. Secondly, hepatic steatosis was assessed using VCTE, which offers greater accuracy compared to ultrasound in detecting hepatic steatosis (60). Thirdly, this study represents the inaugural comparison of the diagnostic efficacy of five commonly utilized NITs—specifically, the FLI, the FSI, the ZJU, the LAP, and the HSI—in the context of MASLD following its recent nomenclature revision. The study provides a comprehensive evaluation of these indices by analyzing their area under the AUC, subgroup AUC, NRI, IDI, and DCA to assess their clinical value. It is important to acknowledge the limitations inherent in this study. Firstly, hepatic steatosis was not diagnosed using the gold standard of hepatic puncture biopsy due to its invasive nature, rendering it impractical for large-scale population screening. Secondly, the study identified variability in the optimal NITs across different cohorts, indicating the necessity for further validation with additional cohorts. Thirdly, there were no data on other rare etiologies that may lead to hepatic steatosis in the two cohorts of this study, such as nutrient deficiency/malnutrition, Wilson’s disease, and celiac disease, which, although these rare etiologies account for a very small percentage of SLD, may still have an impact on the results.

## Conclusion

In the context of MASLD, formerly known as NAFLD, the FLI, the FSI, the ZJU, the LAP, and the HSI demonstrate significant diagnostic utility. Notably, the FSI and FLI exhibit superior diagnostic performance within the United States, whereas the ZJU, FSI, and FLI are more effective within the Chinese population. This finding indicates that different populations may require tailored NITs to achieve optimal diagnostic outcomes. In conclusion, the above NITs are valuable tools for risk screening in MASLD, facilitating the identification of individuals at elevated risk for this condition.

## Data Availability

The datasets presented in this study can be found in online repositories. The names of the repository/repositories and accession number(s) can be found: data pertaining to the NHANES can be accessed on its official website (https://www.cdc.gov/nchs/nhanes/).

## Glossary

NAFLD: non-alcoholic fatty liver disease
MAFLD: metabolic-associated fatty liver disease
SLD: steatotic liver disease
MASLD: metabolic dysfunction associated steatotic liver disease
NITs: non-invasive tests
NCHS: National Center for Health Statistics
NHANES: National Health and Nutrition Examination Survey
CMRF: cardiometabolic risk factor
TRIPOD: multivariable predictive model for individual prognosis or diagnosis
VCTE: vibration-controlled transient elastography
CAP: controlled attenuation parameter
LSM: liver stiffness measurements
IR: insulin resistance
FFAs: free fatty acids
GSH: Glutathione
ROS: reactive oxygen species
BMI: body mass index
WC: waist circumference
ALT: alanine aminotransferase
AST: aspartate aminotransferase
GGT: γ-glutamyl transpeptidase
TG: triglyceride
HDL: high-density lipoprotein cholesterol
ROC: receiver operating characteristic curve
SEN: sensitivity
SPE: specificity
PPV: positive predictive value
NPV: negative predictive value
AUC: area under the receiver operating characteristic curve
NRI: net reclassification index
IDI: integrated discrimination improvement
DCA: decision curve analysis
OR: odds ratio
CI: confidence interval
FLI: fatty liver index
FSI: Framingham steatosis index
ZJU: Zhejiang University index
LAP: lipid accumulation product
HSI: hepatic steatosis index
