# Supplementary material for: Assessment of the diagnostic efficacy of five non-invasive tests for MASLD: external validation utilizing data from two cohorts
Source: Front Nutr. 2025 May 22;12:1571487. doi: 10.3389/fnut.2025.1571487 (PMC12137091; doi:10.3389/fnut.2025.1571487)
Supplement: Supplementary file 1 [file Table_1.docx]

**Assessment of the diagnostic efficacy of five noninvasive tests for MASLD: external validation utilizing data from two distinct cohorts**

**Supplementary material**

1. **Laboratory measurement and clinical data…………………………………………………………...….….2**
2. **Formula for calculating NITs……………………………………………………….………………….……..2**
3. **The definition of MASLD…………………………………………………………………………………..….3**
4. **Referrences………………………………………………………………………………….……………...…..3**
5. **Supplement tables…………………………………………………………………………………….....….….5**

**1.Laboratory measurement and clinical data**

The following variables were obtained from the original NHANES and the Health Management Center at the Hospital of Chengdu Office of the Tibetan Autonomous Region dataset: demographic parameters (age, sex, and race (only for NHANES dataset)), anthropometric parameters (waist circumference (WC), body mass index (BMI)), vibration-controlled transient elastography (VCTE) parameters (liver stiffness measurements (LSM) and controlled attenuation parameter (CAP)), comorbidities (hypertension and diabetes), and biomarkers such as fasting plasma glucose (FPG), triglyceride (TG), alanine aminotransferase (ALT), aspartate aminotransferase (AST), alkaline phosphatase (ALP), γ-glutamyl transpeptidase (GGT), high-density lipoprotein cholesterol (HDL), and total lipoprotein cholesterol (TC). We categorized race into five groups (Hispanic, non-Hispanic Asian, non-Hispanic Black, non-Hispanic White, and other races) (only for NHANES dataset). The diagnostic criteria for diabetes were glycohemoglobin (HbA1c) > 6.5% or random plasma glucose ≥ 11.1 mmol/L or FPG ≥ 7.0 mmol/L or two-hour oral glucose tolerance test (OGTT) plasma glucose ≥ 11.1 mmol/L or under anti-diabetes therapy, or self-reported diabetes [1]. The definition of hypertension was based on systolic blood pressure (SBP) greater than 140 mmHg or diastolic blood pressure (DBP) greater than 90 mmHg, self-reported hypertension, or under antihypertension treatment [2]. The diagnostic criteria for overweight were BMI ≥ 25 kg/m^2^ in NHANES cohort and BMI ≥ 23 in Health Management cohort [3]. Viral hepatitis in the NHANES cohort was defined as the presence of hepatitis C virus infection, indicated by a positive test for viral RNA and/or antibodies, as well as hepatitis B virus infection, indicated by a positive test for surface antigen [4]. A history of amiodarone, methotrexate, tamoxifen, aspirin, ibuprofen, entecavir, protease inhibitors, valproic acid, carbamazepine, fluorouracil, irinotecan, and glucocorticosteroids was defined as a history of medication use that causes hepatic steatosis, according to MASLD-related guidelines [5] (only for NHANES dataset). The calculation of the average weekly alcohol consumption in NHANES cohort was determined by considering the frequency of drinking days within the previous year and the mean quantity of alcohol consumed on those specific days (ALQ121 and ALQ130) [6]. Alcohol intake in the NHANES Alcohol Questionnaire is measured in standard drinks, which we converted to grams based on the conversion standard of 1 standard drink = 14 grams [7]. Excessive alcohol intake was defined as drinking more than 210g of alcohol per week for men and 140g for women both in the NHANES and Health Management Center cohorts [3].

**2.Formula for calculating NITs**

The equations for FLI [8], FSI [9], ZJU [10], ZJU [10], LAP [11], and HSI [12] were as follows: FLI = (e ^0.953 × ln TG (mg/dL) + 0.139 × BMI (kg/m2) + 0.718 × ln GGT (U/L) + 0.053 × WC - 15.745)^/(1 +e ^0.953 × ln TG (mg/dL) + 0.139 × BMI (kg/m2) + 0.718 × ln GGT (U/L) + 0.053 × WC - 15.745)^) × 100; FSI = -7.981 + 0.011 × Age (years) - 0.146 × Sex (female = 1, male = 0) + 0.173 × BMI (kg/m^2^) + 0.007 × TG (mg/dL) + 0.593 × Hypertension (yes = 1, no = 0) + 0.789 × Diabetes (yes = 1, no = 0) + 11 × [ALT (U/L) / AST (U/L) > 1.33 (yes = 1, no = 0)]; ZJU = BMI (kg/m^2^) + FPG (mmol/L) + TG (mmol/L) + 3 × [ALT (U/L) / AST (U/L)] (+ 2, if female); LAP = [WC (cm) - 65] × TG (mmol/L) for males, LAP = [WC (cm) - 58] × TG (mmol/L) for females; HSI = 8 × [ALT (U/L) / AST (U/L)] + BMI (kg/m^2^) (+ 2, if diabetes; + 2, if female).

**3.The definition of MASLD**

MASLD was defined as the presence of hepatic steatosis with one or more of the following [3]:

1) BMI ≥25 kg/m^2^ (≥23 kg/m^2^ for Asia) OR WC >94 cm (for male), 80 cm (for female) OR ethnicity adjusted;

2) FPG ≥5.6 mmol/L OR two-hour OGTT plasma glucose ≥7.8 mmol/L OR HbA1c ≥5.7% OR type 2 diabetes OR treatment for type 2 diabetes;

3) Blood pressure ≥130/85 mmHg OR specific antihypertensive drug treatment;

4) TG ≥1.70 mmol/L OR lipid lowering treatment;

5) HDL ≤1.0 mmol/L for men OR ≤1.3 mmol/L for women OR lipid lowering treatment.

Patients with excessive alcohol consumption and other causes of hepatic steatosis (viral hepatitis and history of taking medications that may cause hepatic steatosis) were excluded, and the diagnostic criteria for heavy excessive consumption were as described above.

**4.Referrences**

1. American Diabetes A: **Classification and Diagnosis of Diabetes: Standards of Medical Care in Diabetes—2020.** *Diabetes Care* 2019, **43:**S14-S31.

2. Williams B, Mancia G, Spiering W, Agabiti Rosei E, Azizi M, Burnier M, Clement DL, Coca A, de Simone G, Dominiczak A, et al: **2018 ESC/ESH Guidelines for the management of arterial hypertension.** *Eur Heart J* 2018, **39:**3021-3104.

3. Rinella ME, Lazarus JV, Ratziu V, Francque SM, Sanyal AJ, Kanwal F, Romero D, Abdelmalek MF, Anstee QM, Arab JP, et al: **A multi-society Delphi consensus statement on new fatty liver disease nomenclature.** *J Hepatol* 2023.

4. Younossi ZM, Stepanova M, Afendy M, Fang Y, Younossi Y, Mir H, Srishord M: **Changes in the prevalence of the most common causes of chronic liver diseases in the United States from 1988 to 2008.** *Clin Gastroenterol Hepatol* 2011, **9:**524-530.e521; quiz e560.

5. **EASL-EASD-EASO Clinical Practice Guidelines on the management of metabolic dysfunction-associated steatotic liver disease (MASLD).** *J Hepatol* 2024, **81:**492-542.

6. **Alcohol Use Questionare** [<https://wwwn.cdc.gov/Nchs/Nhanes/2017-2018/P_ALQ.htm#ALQ121>]

7. **What Is A Standard Drink?** [<https://www.niaaa.nih.gov/alcohols-effects-health/overview-alcohol-consumption/what-standard-drink>]

8. Bedogni G, Bellentani S, Miglioli L, Masutti F, Passalacqua M, Castiglione A, Tiribelli C: **The Fatty Liver Index: a simple and accurate predictor of hepatic steatosis in the general population.** *BMC Gastroenterology* 2006, **6:**33.

9. Long MT, Pedley A, Colantonio LD, Massaro JM, Hoffmann U, Muntner P, Fox CS: **Development and Validation of the Framingham Steatosis Index to Identify Persons With Hepatic Steatosis.** *Clinical Gastroenterology and Hepatology* 2016, **14:**1172-1180.e1172.

10. Wang J, Xu C, Xun Y, Lu Z, Shi J, Yu C, Li YJ: **ZJU index: a novel model for predicting nonalcoholic fatty liver disease in a Chinese population.** *Scientific reports* 2015, **5:**16494.

11. Kahn HS: **The lipid accumulation product is better than BMI for identifying diabetes: a population-based comparison.** *Diabetes Care* 2006, **29:**151-153.

12. Lee J, Kim D, Kim H, Lee C, Yang J, Kim W, Kim Y, Yoon J, Cho S, Sung M, Lee HJ: **Hepatic steatosis index: a simple screening tool reflecting nonalcoholic fatty liver disease.** *Digestive and Liver Disease* 2010, **42:**503-508.

**Supplement Table 1** Performance assessment of the NITs for the prediction of MASLD risk in the NHANES cohort

| Pairwise comparison | NRI (95% CI) | *P* value | IDI (95% CI) | *P* value |
| --- | --- | --- | --- | --- |
| NHANES cohort | | | | |
| FLI vs. FSI | -0.010 (-0.030-0.010) | 0.317 | -0.004 (-0.030-0.010) | 0.729 |
| FLI vs. LAP | 0.033 (0.011-0.055) | 0.003 | 0.037 (0.011-0.055) | 0.002 |
| FLI vs. ZJU | 0.022 (0.004-0.040) | 0.018 | 0.025 (0.004-0.040) | 0.024 |
| FLI vs. HSI | 0.027 (0.007-0.047) | 0.009 | 0.032 (0.007-0.047) | 0.005 |
| FSI vs. LAP | 0.043 (0.021-0.065) | <0.001 | 0.041 (0.021-0.065) | 0.001 |
| FSI vs. ZJU | 0.032 (0.011-0.053) | 0.002 | 0.029 (0.011-0.053) | 0.014 |
| FSI vs. HSI | 0.037 (0.016-0.058) | <0.001 | 0.036 (0.016-0.058) | 0.002 |
| LAP vs. ZJU | -0.010 (-0.034-0.014) | 0.421 | -0.012 (-0.034-0.014) | 0.338 |
| LAP vs. HSI | -0.006 (-0.032-0.020) | 0.651 | -0.005 (-0.032-0.020) | 0.699 |
| ZJU vs. HSI | 0.004 (-0.011-0.019) | 0.607 | 0.007 (-0.011-0.019) | 0.480 |
| Health Management Center cohort | | | | |
| FLI vs. FSI | 0.010 (-0.026-0.046) | 0.591 | 0.011 (-0.026-0.048) | 0.561 |
| FLI vs. LAP | 0.050 (0.019-0.081) | 0.002 | 0.047 (0.013-0.081) | 0.006 |
| FLI vs. ZJU | -0.009 (-0.047-0.029) | 0.646 | -0.005 (-0.044-0.034) | 0.801 |
| FLI vs. HSI | 0.021 (-0.020-0.062) | 0.312 | 0.023 (-0.016-0.062) | 0.253 |
| FSI vs. LAP | 0.040 (0.002-0.078) | 0.041 | 0.036 (-0.002-0.074) | 0.060 |
| FSI vs. ZJU | -0.019 (-0.054-0.016) | 0.281 | -0.016 (-0.053-0.021) | 0.395 |
| FSI vs. HSI | 0.010 (-0.026-0.046) | 0.582 | 0.011 (-0.026-0.048) | 0.558 |
| LAP vs. ZJU | -0.059 (-0.100--0.018) | 0.005 | -0.052 (-0.091--0.013) | 0.010 |
| LAP vs. HSI | -0.030 (-0.075-0.015) | 0.194 | -0.024 (-0.065-0.017) | 0.247 |
| ZJU vs. HSI | 0.030 (0.001-0.059) | 0.043 | 0.027 (-0.007-0.130) | 0.115 |

Note: NRI, net reclassification index; IDI, integrated discrimination improvement.

**Supplement Table** **2** Subgroup analysis of FLI for predicting MASLD risk in the NHANES cohort

| Subgroup | AUC  (95% CI) | SEN  (95% CI) | SPE  (95% CI) | PPV  (95% CI) | NPV  (95% CI) | Cutoff value |
| --- | --- | --- | --- | --- | --- | --- |
| Sex | | | | | | |
| Female | 0.821  (0.806-0.837) | 0.839  (0.817-0.861) | 0.654  (0.631-0.677) | 0.602  (0.577-0.627) | 0.867  (0.848-0.886) | 48.427 |
| Male | 0.849  (0.835-0.862) | 0.789  (0.767-0.811) | 0.735  (0.713-0.758) | 0.735  (0.713-0.758) | 0.789  (0.767-0.811) | 62.102 |
| Race |  |  |  |  |  |  |
| Black | 0.838  (0.817-0.858) | 0.780  (0.744-0.817) | 0.742  (0.712-0.771) | 0.632  (0.594-0.671) | 0.856  (0.830-0.881) | 71.545 |
| White | 0.853  (0.837-0.869) | 0.779  (0.752-0.806) | 0.755  (0.730-0.780) | 0.715  (0.687-0.743) | 0.812  (0.789-0.836) | 63.920 |
| Hispanic | 0.824  (0.802-0.846) | 0.754  (0.722-0.787) | 0.720  (0.685-0.755) | 0.741  (0.708-0.774) | 0.734  (0.700-0.769) | 62.676 |
| Asian | 0.842  (0.810-0.875) | 0.804  (0.751-0.856) | 0.741  (0.695-0.788) | 0.664  (0.607-0.721) | 0.856  (0.816-0.896) | 40.719 |
| Other | 0.832  (0.785-0.879) | 0.815  (0.745-0.885) | 0.712  (0.640-0.783) | 0.683  (0.607-0.760) | 0.835  (0.771-0.898) | 62.627 |
| Age | | | | | | |
| <60 | 0.857  (0.845-0.869) | 0.819  (0.799-0.839) | 0.724  (0.705-0.742) | 0.664  (0.642-0.685) | 0.857  (0.841-0.873) | 55.392 |
| ≥60 | 0.784  (0.764-0.805) | 0.738  (0.710-0.767) | 0.682  (0.652-0.712) | 0.701  (0.672-0.730) | 0.721  (0.691-0.751) | 62.857 |
| Overweight | | | | | | |
| No | 0.813  (0.776-0.850) | 0.787  (0.710-0.864) | 0.693  (0.667-0.719) | 0.189  (0.153-0.226) | 0.973  (0.962-0.984) | 15.558 |
| Yes | 0.771  (0.757-0.785) | 0.642  (0.623-0.662) | 0.761  (0.742-0.780) | 0.760  (0.741-0.779) | 0.644  (0.624-0.663) | 78.603 |
| Hypertension | | | | | | |
| No | 0.843  (0.830-0.855) | 0.867  (0.848-0.885) | 0.658  (0.638-0.677) | 0.584  (0.562-0.607) | 0.899  (0.884-0.913) | 43.596 |
| Yes | 0.791  (0.771-0.811) | 0.688  (0.661-0.715) | 0.748  (0.718-0.777) | 0.786  (0.760-0.812) | 0.640  (0.610-0.670) | 77.641 |
| Diabetes | | | | | | |
| No | 0.834  (0.822-0.846) | 0.835  (0.817-0.853) | 0.674  (0.657-0.691) | 0.605  (0.585-0.625) | 0.872  (0.853-0.674) | 48.693 |
| Yes | 0.766  (0.735-0.797) | 0.613  (0.577-0.648) | 0.769  (0.724-0.815) | 0.852  (0.821-0.882) | 0.479  (0.436-0.521) | 85.934 |

Note: SEN, sensitivity; SPE, speciﬁcity; PPV, positive predictive value; NPV, negative predictive value; AUC, area under receiver operating characteristic curve. Black means non-Hispanic Black, White means non-Hispanic White, Asian means non-Hispanic Asian, and Other means other races.

**Supplement Table** **3** Subgroup analysis of FSI for predicting MASLD risk in the NHANES cohort

| Subgroup | AUC  (95% CI) | SEN  (95% CI) | SPE  (95% CI) | PPV  (95% CI) | NPV  (95% CI) | Cutoff value |
| --- | --- | --- | --- | --- | --- | --- |
| Sex | | | | | | |
| Female | 0.822  (0.806-0.837) | 0.788  (0.763-0.812) | 0.701  (0.679-0.723) | 0.621  (0.595-0.647) | 0.841  (0.822-0.860) | -1.154 |
| Male | 0.849  (0.835-0.863) | 0.796  (0.775-0.818) | 0.743  (0.720-0.765) | 0.743  (0.720-0.765) | 0.796  (0.775-0.818) | -0.942 |
| Race |  |  |  |  |  |  |
| Black | 0.831  (0.810-0.853) | 0.801  (0.765-0.836) | 0.731  (0.701-0.761) | 0.629  (0.591-0.667) | 0.866  (0.841-0.891) | -0.761 |
| White | 0.848  (0.832-0.865) | 0.806  (0.781-0.832) | 0.725  (0.700-0.751) | 0.698  (0.671-0.726) | 0.826  (0.803-0.850) | -1.068 |
| Hispanic | 0.820  (0.798-0.842) | 0.784  (0.753-0.815) | 0.690  (0.654-0.726) | 0.729  (0.697-0.761) | 0.751  (0.716-0.786) | -1.063 |
| Asian | 0.857  (0.826-0.888) | 0.817  (0.766-0.869) | 0.767  (0.723-0.812) | 0.691  (0.635-0.747) | 0.868  (0.830-0.906) | -1.522 |
| Other | 0.852  (0.807-0.896) | 0.714  (0.633-0.795) | 0.853  (0.797-0.908) | 0.787  (0.710-0.864) | 0.796  (0.735-0.857) | -0.411 |
| Age | | | | | | |
| <60 | 0.863  (0.851-0.874) | 0.767  (0.746-0.789) | 0.794  (0.777-0.811) | 0.712  (0.690-0.735) | 0.837  (0.821-0.853) | -0.974 |
| ≥60 | 0.770  (0.748-0.791) | 0.729  (0.700-0.757) | 0.682  (0.652-0.712) | 0.698  (0.669-0.727) | 0.713  (0.683-0.743) | -0.656 |
| Overweight | | | | | | |
| No | 0.796  (0.756-0.836) | 0.843  (0.774-0.911) | 0.614  (0.587-0.642) | 0.166  (0.135-0.197) | 0.977  (0.966-0.988) | -2.868 |
| Yes | 0.774  (0.760-0.788) | 0.694  (0.676-0.713) | 0.721  (0.701-0.741) | 0.746  (0.727-0.764) | 0.667  (0.647-0.687) | -0.466 |
| Hypertension | | | | | | |
| No | 0.844  (0.831-0.857) | 0.786  (0.763-0.808) | 0.733  (0.715-0.751) | 0.620  (0.597-0.644) | 0.860  (0.845-0.876) | -1.470 |
| Yes | 0.793  (0.773-0.812) | 0.651  (0.623-0.679) | 0.782  (0.754-0.810) | 0.801  (0.775-0.827) | 0.625  (0.595-0.654) | 0.204 |
| Diabetes | | | | | | |
| No | 0.829  (0.818-0.841) | 0.721  (0.699-0.742) | 0.771  (0.755-0.876) | 0.653  (0.631-0.675) | 0.822  (0.807-0.836) | -1.068 |
| Yes | 0.770  (0.739-0.800) | 0.663  (0.628-0.697) | 0.743  (0.696-0.789) | 0.848  (0.818-0.877) | 0.504  (0.460-0.548) | 0.598 |

Note: SEN, sensitivity; SPE, speciﬁcity; PPV, positive predictive value; NPV, negative predictive value; AUC, area under receiver operating characteristic curve. Black means non-Hispanic Black, White means non-Hispanic White, Asian means non-Hispanic Asian, and Other means other races.

**Supplement Table 4** Subgroup analysis of LAP for predicting MASLD risk in the NHANES cohort

| Subgroup | AUC  (95% CI) | SEN  (95% CI) | SPE  (95% CI) | PPV  (95% CI) | NPV  (95% CI) | Cutoff value |
| --- | --- | --- | --- | --- | --- | --- |
| Sex | | | | | | |
| Female | 0.802  (0.786-0.818) | 0.747  (0.720-0.773) | 0.712  (0.690-0.733) | 0.617  (0.591-0.644) | 0.818  (0.799-0.838) | 52.376 |
| Male | 0.825  (0.810-0.840) | 0.749  (0.726-0.772) | 0.742  (0.719-0.764) | 0.730  (0.706-0.753) | 0.760  (0.738-0.782) | 50.447 |
| Race |  |  |  |  |  |  |
| Black | 0.810  (0.787-0.832) | 0.854  (0.823-0.886) | 0.640  (0.608-0.672) | 0.575  (0.539-0.611) | 0.885  (0.860-0.910) | 37.837 |
| White | 0.820  (0.802-0.838) | 0.766  (0.739-0.794) | 0.736  (0.710-0.762) | 0.696  (0.667-0.725) | 0.800  (0.776-0.824) | 55.345 |
| Hispanic | 0.787  (0.762-0.811) | 0.701  (0.666-0.736) | 0.728  (0.694-0.763) | 0.733  (0.698-0.767) | 0.696  (0.661-0.731) | 56.596 |
| Asian | 0.833  (0.800-0.866) | 0.712  (0.652-0.772) | 0.791  (0.748-0.834) | 0.684  (0.624-0.745) | 0.812  (0.770-0.854) | 47.623 |
| Other | 0.806  (0.756-0.857) | 0.874  (0.814-0.934) | 0.603  (0.526-0.679) | 0.627  (0.553-0.700) | 0.862  (0.798-0.927) | 38.257 |
| Age | | | | | | |
| <60 | 0.834  (0.821-0.847) | 0.756  (0.734-0.778) | 0.753  (0.735-0.771) | 0.670  (0.648-0.693) | 0.822  (0.806-0.839) | 50.004 |
| ≥60 | 0.760  (0.738-0.781) | 0.683  (0.653-0.713) | 0.717  (0.688-0.746) | 0.709  (0.679-0.739) | 0.691  (0.662-0.721) | 59.130 |
| Overweight | | | | | | |
| No | 0.812  (0.776-0.849) | 0.778  (0.699-0.856) | 0.714  (0.688-0.740) | 0.199  (0.161-0.237) | 0.972  (0.962-0.983) | 23.400 |
| Yes | 0.745  (0.730-0.760) | 0.706  (0.687-0.725) | 0.664  (0.643-0.685) | 0.712  (0.693-0.731) | 0.657  (0.636-0.678) | 57.061 |
| Hypertension | | | | | | |
| No | 0.820  (0.807-0.834) | 0.719  (0.695-0.744) | 0.762  (0.745-0.780) | 0.627  (0.602-0.652) | 0.830  (0.814-0.846) | 50.111 |
| Yes | 0.770  (0.749-0.791) | 0.741  (0.716-0.767) | 0.663  (0.631-0.696) | 0.748  (0.722-0.774) | 0.656  (0.623-0.688) | 56.722 |
| Diabetes | | | | | | |
| No | 0.810  (0.797-0.822) | 0.757  (0.737-0.778) | 0.707  (0.690-0.724) | 0.607  (0.586-0.628) | 0.829  (0.814-0.845) | 46.434 |
| Yes | 0.742  (0.710-0.774) | 0.620  (0.584-0.655) | 0.760  (0.715-0.806) | 0.848  (0.818-0.879) | 0.480  (0.438-0.523) | 74.790 |

Note: SEN, sensitivity; SPE, speciﬁcity; PPV, positive predictive value; NPV, negative predictive value; AUC, area under receiver operating characteristic curve. Black means non-Hispanic Black, White means non-Hispanic White, Asian means non-Hispanic Asian, and Other means other races.

**Supplement Table 5** Subgroup analysis of ZJU for predicting MASLD risk in the NHANES cohort

| Subgroup | AUC  (95% CI) | SEN  (95% CI) | SPE  (95% CI) | PPV  (95% CI) | NPV  (95% CI) | Cutoff value |
| --- | --- | --- | --- | --- | --- | --- |
| Sex | | | | | | |
| Female | 0.810  (0.794-0.826) | 0.875  (0.855-0.895) | 0.585  (0.561-0.608) | 0.568  (0.544-0.592) | 0.883  (0.864-0.902) | 38.873 |
| Male | 0.844  (0.830-0.858) | 0.755  (0.732-0.778) | 0.774  (0.753-0.796) | 0.757  (0.734-0.780) | 0.772  (0.750-0.794) | 39.495 |
| Race |  |  |  |  |  |  |
| Black | 0.814  (0.791-0.836) | 0.846  (0.814-0.878) | 0.637  (0.605-0.670) | 0.571  (0.535-0.607) | 0.879  (0.853-0.905) | 40.192 |
| White | 0.827  (0.810-0.845) | 0.742  (0.713-0.771) | 0.761  (0.737-0.786) | 0.710  (0.681-0.739) | 0.789  (0.765-0.813) | 40.497 |
| Hispanic | 0.815  (0.792-0.837) | 0.771  (0.739-0.803) | 0.700  (0.664-0.736) | 0.732  (0.699-0.764) | 0.742  (0.707-0.777) | 40.352 |
| Asian | 0.840  (0.807-0.873) | 0.817  (0.766-0.869) | 0.735  (0.689-0.782) | 0.663  (0.607-0.719) | 0.863  (0.824-0.903) | 36.173 |
| Other | 0.816  (0.767-0.866) | 0.882  (0.824-0.940) | 0.635  (0.559-0.710) | 0.648  (0.575-0.722) | 0.876  (0.815-0.937) | 38.353 |
| Age | | | | | | |
| <60 | 0.844  (0.832-0.857) | 0.855  (0.837-0.873) | 0.672  (0.652-0.691) | 0.634  (0.613-0.655) | 0.874  (0.859-0.890) | 38.848 |
| ≥60 | 0.756  (0.735-0.778) | 0.799  (0.773-0.825) | 0.595  (0.564-0.627) | 0.666  (0.638-0.694) | 0.746  (0.714-0.778) | 38.504 |
| Overweight | | | | | | |
| No | 0.779  (0.739-0.819) | 0.824  (0.752-0.896) | 0.635  (0.608-0.663) | 0.171  (0.139-0.203) | 0.975  (0.964-0.986) | 32.259 |
| Yes | 0.742  (0.728-0.757) | 0.634  (0.614-0.654) | 0.723  (0.703-0.743) | 0.730  (0.710-0.749) | 0.627  (0.607-0.647) | 42.963 |
| Hypertension | | | | | | |
| No | 0.824  (0.811-0.838) | 0.795  (0.772-0.817) | 0.701  (0.682-0.720) | 0.596  (0.573-0.620) | 0.860  (0.844-0.876) | 38.848 |
| Yes | 0.771  (0.750-0.792) | 0.788  (0.764-0.812) | 0.627  (0.595-0.660) | 0.740  (0.715-0.765) | 0.687  (0.654-0.720) | 40.470 |
| Diabetes | | | | | | |
| No | 0.811  (0.798-0.823) | 0.802  (0.782-0.821) | 0.672  (0.654-0.689) | 0.594  (0.574-0.614) | 0.850  (0.835-0.865) | 38.538 |
| Yes | 0.744  (0.711-0.777) | 0.667  (0.632-0.701) | 0.716  (0.667-0.764) | 0.835  (0.805-0.866) | 0.498  (0.453-0.543) | 44.417 |

Note: SEN, sensitivity; SPE, speciﬁcity; PPV, positive predictive value; NPV, negative predictive value; AUC, area under receiver operating characteristic curve. Black means non-Hispanic Black, White means non-Hispanic White, Asian means non-Hispanic Asian, and Other means other races.

**Supplement Table 6** Subgroup analysis of HSI for predicting MASLD risk in the NHANES cohort

| Subgroup | AUC  (95% CI) | SEN  (95% CI) | SPE  (95% CI) | PPV  (95% CI) | NPV  (95% CI) | Cutoff value |
| --- | --- | --- | --- | --- | --- | --- |
| Sex | | | | | | |
| Female | 0.805  (0.789-0.821) | 0.752  (0.726-0.778) | 0.706  (0.684-0.727) | 0.614  (0.588-0.641) | 0.821  (0.801-0.840) | 39.784 |
| Male | 0.835  (0.821-0.850) | 0.746  (0.723-0.769) | 0.770  (0.748-0.792) | 0.751  (0.728-0.775) | 0.765  (0.743-0.786) | 38.232 |
| Race |  |  |  |  |  |  |
| Black | 0.818  (0.795-0.840) | 0.817  (0.783-0.852) | 0.670  (0.639-0.702) | 0.585  (0.548-0.622) | 0.866  (0.840-0.892) | 39.480 |
| White | 0.816  (0.798-0.835) | 0.754  (0.726-0.782) | 0.732  (0.707-0.758) | 0.690  (0.661-0.719) | 0.791  (0.766-0.815) | 38.367 |
| Hispanic | 0.813  (0.790-0.836) | 0.804  (0.774-0.834) | 0.679  (0.643-0.716) | 0.727  (0.695-0.759) | 0.765  (0.730-0.800) | 38.212 |
| Asian | 0.823  (0.789-0.858) | 0.699  (0.638-0.759) | 0.823  (0.782-0.863) | 0.715  (0.654-0.775) | 0.811  (0.770-0.852) | 36.230 |
| Other | 0.820  (0.770-0.869) | 0.782  (0.707-0.856) | 0.750  (0.682-0.818) | 0.705  (0.627-0.782) | 0.818  (0.755-0.881) | 39.831 |
| Age | | | | | | |
| <60 | 0.843  (0.831-0.855) | 0.822  (0.802-0.841) | 0.707  (0.688-0.726) | 0.651  (0.630-0.673) | 0.856  (0.840-0.872) | 38.291 |
| ≥60 | 0.754  (0.732-0.776) | 0.745  (0.717-0.773) | 0.648  (0.617-0.679) | 0.681  (0.652-0.710) | 0.715  (0.685-0.746) | 37.350 |
| Overweight | | | | | | |
| No | 0.725  (0.680-0.769) | 0.852  (0.785-0.919) | 0.484  (0.456-0.513) | 0.131  (0.106-0.156) | 0.973  (0.960-0.986) | 29.281 |
| Yes | 0.739  (0.724-0.754) | 0.640  (0.621-0.660) | 0.715  (0.695-0.735) | 0.726  (0.707-0.745) | 0.628  (0.608-0.648) | 41.426 |
| Hypertension | | | | | | |
| No | 0.820  (0.806-0.834) | 0.797  (0.775-0.819) | 0.693  (0.675-0.712) | 0.591  (0.567-0.614) | 0.860  (0.844-0.876) | 37.126 |
| Yes | 0.769  (0.748-0.790) | 0.757  (0.732-0.782) | 0.666  (0.634-0.698) | 0.753  (0.728-0.778) | 0.671  (0.639-0.703) | 39.495 |
| Diabetes | | | | | | |
| No | 0.805  (0.792-0.817) | 0.749  (0.728-0.770) | 0.710  (0.693-0.727) | 0.607  (0.586-0.628) | 0.825  (0.810-0.840) | 37.854 |
| Yes | 0.753  (0.721-0.784) | 0.819  (0.791-0.847) | 0.557  (0.504-0.610) | 0.800  (0.771-0.829) | 0.587  (0.533-0.641) | 39.689 |

Note: SEN, sensitivity; SPE, speciﬁcity; PPV, positive predictive value; NPV, negative predictive value; AUC, area under receiver operating characteristic curve. Black means non-Hispanic Black, White means non-Hispanic White, Asian means non-Hispanic Asian, and Other means other races.

**Supplement Table 7** Subgroup analysis of FLI for predicting MASLD risk in the Health Management Center cohort

| Subgroup | AUC  (95% CI) | SEN  (95% CI) | SPE  (95% CI) | PPV  (95% CI) | NPV  (95% CI) | Cutoff value |
| --- | --- | --- | --- | --- | --- | --- |
| Sex | | | | | | |
| Female | 0.834  (0.806-0.863) | 0.850  (0.802-0.899) | 0.684  (0.645-0.722) | 0.501  (0.449-0.554) | 0.924  (0.899-0.950) | 22.930 |
| Male | 0.793  (0.770-0.816) | 0.760  (0.726-0.793) | 0.701  (0.669-0.733) | 0.666  (0.632-0.701) | 0.788  (0.758-0.818) | 53.087 |
| Age | | | | | | |
| <60 | 0.819  (0.801-0.838) | 0.865  (0.840-0.890) | 0.633  (0.606-0.660) | 0.585  (0.555-0.615) | 0.887  (0.865-0.908) | 35.727 |
| ≥60 | 0.733  (0.673-0.792) | 0.712  (0.630-0.794) | 0.667  (0.592-0.741) | 0.622  (0.540-0.704) | 0.750  (0.677-0.823) | 46.963 |
| Overweight | | | | | | |
| No | 0.809  (0.739-0.879) | 0.926  (0.827-1.000) | 0.603  (0.558-0.648) | 0.120  (0.076-0.164) | 0.990  (0.980-1.000) | 12.562 |
| Yes | 0.743  (0.720-0.766) | 0.689  (0.657-0.721) | 0.686  (0.655-0.716) | 0.665  (0.634-0.697) | 0.709  (0.678-0.739) | 52.516 |
| Hypertension | | | | | | |
| No | 0.814  (0.794-0.833) | 0.844  (0.815-0.872) | 0.644  (0.617-0.671) | 0.556  (0.525-0.588) | 0.886  (0.865-0.907) | 35.727 |
| Yes | 0.738  (0.686-0.790) | 0.871  (0.825-0.917) | 0.494  (0.415-0.572) | 0.690  (0.633-0.747) | 0.748  (0.664-0.831) | 43.086 |
| Diabetes | | | | | | |
| No | 0.806  (0.787-0.825) | 0.850  (0.825-0.876) | 0.625  (0.599-0.652) | 0.564  (0.534-0.593) | 0.880  (0.859-0.901) | 35.899 |
| Yes | 0.805  (0.733-0.876) | 0.780  (0.699-0.861) | 0.721  (0.609-0.834) | 0.821  (0.744-0.898) | 0.667  (0.553-0.780) | 54.045 |

Note: SEN, sensitivity; SPE, speciﬁcity; PPV, positive predictive value; NPV, negative predictive value; AUC, area under receiver operating characteristic curve. Black means non-Hispanic Black, White means non-Hispanic White, Asian means non-Hispanic Asian, and Other means other races.

**Supplement Table** **8** Subgroup analysis of FSI for predicting MASLD risk in the Health Management Center cohort

| Subgroup | AUC  (95% CI) | SEN  (95% CI) | SPE  (95% CI) | PPV  (95% CI) | NPV  (95% CI) | Cutoff value |
| --- | --- | --- | --- | --- | --- | --- |
| Sex | | | | | | |
| Female | 0.846  (0.818-0.874) | 0.845  (0.796-0.895) | 0.703  (0.665-0.742) | 0.516  (0.463-0.569) | 0.924  (0.899-0.949) | -2.125 |
| Male | 0.773  (0.749-0.797) | 0.745  (0.711-0.779) | 0.688  (0.656-0.720) | 0.653  (0.618-0.688) | 0.775  (0.744-0.805) | -1.141 |
| Age | | | | | | |
| <60 | 0.809  (0.789-0.828) | 0.734  (0.701-0.766) | 0.741  (0.717-0.766) | 0.629  (0.596-0.662) | 0.823  (0.800-0.846) | -1.307 |
| ≥60 | 0.781  (0.728-0.835) | 0.881  (0.823-0.940) | 0.575  (0.497-0.653) | 0.615  (0.542-0.689) | 0.863  (0.796-0.930) | -1.465 |
| Overweight | | | | | | |
| No | 0.771  (0.706-0.835) | 0.963  (0.892-1.000) | 0.566  (0.521-0.611) | 0.115  (0.073-0.157) | 0.992  (0.985-1.000) | -2.877 |
| Yes | 0.743  (0.720-0.766) | 0.705  (0.674-0.737) | 0.671  (0.640-0.702) | 0.660  (0.629-0.692) | 0.715  (0.685-0.746) | -1.141 |
| Hypertension | | | | | | |
| No | 0.806  (0.786-0.826) | 0.769  (0.737-0.802) | 0.702  (0.676-0.727) | 0.577  (0.544-0.610) | 0.852  (0.830-0.874) | -1.521 |
| Yes | 0.748  (0.698-0.799) | 0.649  (0.583-0.714) | 0.724  (0.654-0.794) | 0.753  (0.689-0.817) | 0.614  (0.544-0.684) | -0.358 |
| Diabetes | | | | | | |
| No | 0.802  (0.783-0.821) | 0.714  (0.682-0.747) | 0.748  (0.725-0.772) | 0.618  (0.585-0.650) | 0.821  (0.800-0.843) | -1.307 |
| Yes | 0.774  (0.699-0.848) | 0.900  (0.841-0.959) | 0.525  (0.399-0.650) | 0.756  (0.679-0.833) | 0.762  (0.633-0.891) | -0.790 |

Note: SEN, sensitivity; SPE, speciﬁcity; PPV, positive predictive value; NPV, negative predictive value; AUC, area under receiver operating characteristic curve. Black means non-Hispanic Black, White means non-Hispanic White, Asian means non-Hispanic Asian, and Other means other races.

**Supplement Table 9** Subgroup analysis of LAP for predicting MASLD risk in the Health Management Center cohort

| Subgroup | AUC  (95% CI) | SEN  (95% CI) | SPE  (95% CI) | PPV  (95% CI) | NPV  (95% CI) | Cutoff value |
| --- | --- | --- | --- | --- | --- | --- |
| Sex | | | | | | |
| Female | 0.818  (0.787-0.848) | 0.894  (0.852-0.936) | 0.620  (0.580-0.661) | 0.468  (0.419-0.518) | 0.940  (0.915-0.964) | 23.245 |
| Male | 0.748  (0.723-0.773) | 0.682  (0.645-0.718) | 0.700  (0.668-0.731) | 0.641  (0.604-0.677) | 0.736  (0.705-0.768) | 40.580 |
| Age | | | | | | |
| <60 | 0.787  (0.767-0.807) | 0.792  (0.762-0.822) | 0.655  (0.628-0.682) | 0.578  (0.548-0.609) | 0.840  (0.817-0.864) | 31.810 |
| ≥60 | 0.716  (0.655-0.776) | 0.669  (0.585-0.754) | 0.686  (0.613-0.760) | 0.622  (0.538-0.706) | 0.729  (0.657-0.802) | 40.410 |
| Overweight | | | | | | |
| No | 0.839  (0.763-0.915) | 0.889  (0.770-1.000) | 0.740  (0.700-0.780) | 0.167  (0.106-0.228) | 0.990  (0.980-1.000) | 17.720 |
| Yes | 0.709  (0.685-0.733) | 0.726  (0.696-0.757) | 0.589  (0.557-0.622) | 0.616  (0.585-0.647) | 0.704  (0.671-0.737) | 36.660 |
| Hypertension | | | | | | |
| No | 0.783  (0.762-0.804) | 0.784  (0.751-0.816) | 0.660  (0.633-0.687) | 0.549  (0.517-0.582) | 0.852  (0.829-0.875) | 31.810 |
| Yes | 0.709  (0.656-0.763) | 0.569  (0.501-0.638) | 0.744  (0.675-0.812) | 0.742  (0.673-0.811) | 0.571  (0.503-0.640) | 51.490 |
| Diabetes | | | | | | |
| No | 0.776  (0.755-0.796) | 0.780  (0.750-0.810) | 0.643  (0.617-0.669) | 0.554  (0.524-0.584) | 0.837  (0.814-0.860) | 31.810 |
| Yes | 0.751  (0.673-0.829) | 0.930  (0.880-0.980) | 0.459  (0.334-0.584) | 0.738  (0.661-0.815) | 0.800  (0.667-0.933) | 31.885 |

Note: SEN, sensitivity; SPE, speciﬁcity; PPV, positive predictive value; NPV, negative predictive value; AUC, area under receiver operating characteristic curve.

**Supplement Table 10** Subgroup analysis of ZJU for predicting MASLD risk in the Health Management Center cohort

| Subgroup | AUC  (95% CI) | SEN  (95% CI) | SPE  (95% CI) | PPV  (95% CI) | NPV  (95% CI) | Cutoff value |
| --- | --- | --- | --- | --- | --- | --- |
| Sex | | | | | | |
| Female | 0.841  (0.812-0.869) | 0.758  (0.700-0.817) | 0.794  (0.760-0.828) | 0.579  (0.521-0.638) | 0.898  (0.871-0.925) | 38.151 |
| Male | 0.803  (0.781-0.825) | 0.774  (0.741-0.807) | 0.703  (0.672-0.735) | 0.672  (0.638-0.706) | 0.798  (0.769-0.828) | 37.171 |
| Age | | | | | | |
| <60 | 0.820  (0.801-0.838) | 0.775  (0.745-0.806) | 0.721  (0.695-0.746) | 0.624  (0.592-0.656) | 0.843  (0.821-0.865) | 37.174 |
| ≥60 | 0.774  (0.719-0.829) | 0.814  (0.743-0.884) | 0.634  (0.558-0.710) | 0.632  (0.555-0.708) | 0.815  (0.745-0.885) | 37.151 |
| Overweight | | | | | | |
| No | 0.844  (0.775-0.912) | 0.926  (0.827-1.000) | 0.633  (0.589-0.677) | 0.129  (0.082-0.176) | 0.990  (0.981-1.000) | 32.779 |
| Yes | 0.748  (0.725-0.771) | 0.726  (0.696-0.757) | 0.660  (0.629-0.691) | 0.660  (0.628-0.691) | 0.727  (0.696-0.758) | 37.994 |
| Hypertension | | | | | | |
| No | 0.817  (0.798-0.837) | 0.761  (0.728-0.795) | 0.729  (0.704-0.754) | 0.598  (0.564-0.632) | 0.852  (0.831-0.874) | 37.174 |
| Yes | 0.762  (0.712-0.812) | 0.827  (0.775-0.879) | 0.590  (0.513-0.667) | 0.723  (0.665-0.781) | 0.724  (0.647-0.802) | 37.288 |
| Diabetes | | | | | | |
| No | 0.814  (0.796-0.833) | 0.756  (0.725-0.787) | 0.730  (0.705-0.754) | 0.614  (0.583-0.646) | 0.840  (0.819-0.862) | 37.174 |
| Yes | 0.742  (0.663-0.822) | 0.790  (0.710-0.870) | 0.639  (0.519-0.760) | 0.782  (0.702-0.863) | 0.650  (0.529-0.771) | 40.468 |

Note: SEN, sensitivity; SPE, speciﬁcity; PPV, positive predictive value; NPV, negative predictive value; AUC, area under receiver operating characteristic curve.

**Supplement Table 11** Subgroup analysis of HSI for predicting MASLD risk in the Health Management Center cohort

| Subgroup | AUC  (95% CI) | SEN  (95% CI) | SPE  (95% CI) | PPV  (95% CI) | NPV  (95% CI) | Cutoff value |
| --- | --- | --- | --- | --- | --- | --- |
| Sex | | | | | | |
| Female | 0.812  (0.779-0.844) | 0.758  (0.700-0.817) | 0.738  (0.701-0.774) | 0.520  (0.464-0.576) | 0.891  (0.862-0.919) | 36.975 |
| Male | 0.774  (0.750-0.798) | 0.745  (0.711-0.779) | 0.688  (0.656-0.720) | 0.653  (0.618-0.688) | 0.775  (0.744-0.805) | 37.694 |
| Age | | | | | | |
| <60 | 0.799  (0.780-0.819) | 0.799  (0.770-0.828) | 0.679  (0.652-0.705) | 0.598  (0.567-0.629) | 0.850  (0.827-0.872) | 36.824 |
| ≥60 | 0.738  (0.679-0.796) | 0.856  (0.793-0.919) | 0.536  (0.457-0.615) | 0.587  (0.514-0.661) | 0.828  (0.754-0.903) | 35.118 |
| Overweight | | | | | | |
| No | 0.733  (0.654-0.813) | 0.741  (0.575-0.906) | 0.692  (0.650-0.734) | 0.123  (0.073-0.174) | 0.979  (0.963-0.994) | 32.435 |
| Yes | 0.721  (0.697-0.745) | 0.762  (0.733-0.792) | 0.585  (0.552-0.617) | 0.625  (0.595-0.655) | 0.731  (0.698-0.763) | 37.640 |
| Hypertension | | | | | | |
| No | 0.794  (0.773-0.815) | 0.785  (0.753-0.817) | 0.687  (0.661-0.714) | 0.571  (0.538-0.603) | 0.858  (0.836-0.880) | 36.892 |
| Yes | 0.759  (0.709-0.809) | 0.748  (0.688-0.807) | 0.647  (0.572-0.722) | 0.733  (0.673-0.793) | 0.664  (0.589-0.740) | 37.750 |
| Diabetes | | | | | | |
| No | 0.789  (0.769-0.808) | 0.763  (0.733-0.794) | 0.692  (0.667-0.718) | 0.586  (0.554-0.617) | 0.837  (0.815-0.859) | 36.956 |
| Yes | 0.759  (0.681-0.837) | 0.870  (0.804-0.936) | 0.541  (0.416-0.666) | 0.757  (0.678-0.835) | 0.717  (0.587-0.848) | 37.128 |

Note: SEN, sensitivity; SPE, speciﬁcity; PPV, positive predictive value; NPV, negative predictive value; AUC, area under receiver operating characteristic curve.
